# Effect of partially hydrolyzed guar gum on the expression of aquaporin‐3 in the colon

**DOI:** 10.1002/fsn3.3150

**Published:** 2022-11-17

**Authors:** Risako Kon, Nobutomo Ikarashi, Kazuhiro Onuma, Zenta Yasukawa, Makoto Ozeki, Hiroyasu Sakai, Junzo Kamei

**Affiliations:** ^1^ Department of Biomolecular Pharmacology Hoshi University Tokyo Japan; ^2^ Department of Nutrition, Faculty of Nutrition Kanazawa Gakuin University Ishikawa Japan; ^3^ Taiyo Kagaku Co., Ltd. Mie Japan; ^4^ Advanced Research Institute for Health Science Juntendo University Tokyo Japan

**Keywords:** aquaporin, colon, constipation, fiber, partially hydrolyzed guar gum

## Abstract

In recent years, the development of functional foods targeting gastrointestinal disorders has been in progress. Partially hydrolyzed guar gum (PHGG), which is a water‐soluble dietary fiber, is known to have a constipation‐improving effect. However, many aspects of the mechanism remain unclear. In this study, we investigated the role of aquaporin‐3 (AQP3), which regulates the water content of feces in ameliorative effect of PHGG on constipation. Rats were allowed to freely consume a normal diet or a diet containing 5% PHGG for 14 days, and defecation parameters were measured. We also analyzed the expression levels of genes involved in water transport in the colon. The defecation frequency and volume of rats treated with PHGG were not different from those from the control group, but the fecal water content was significantly increased, and soft stools were observed. The expressions of claudin‐1, tight junction protein‐1, and cadherin‐1, which are involved in tight junctions or adherens junctions, were almost the same in the PHGG‐treated group and the control group. The expression level of AQP3 in the colon was significantly decreased in the PHGG‐treated group. In this study, PHGG decreased the colonic AQP3 expression, thereby suppressing water transport from the luminal side to the vascular side and improving constipation.

## Graphical abstract

We found that PHGG decreases the expression level of AQP3 without changing the osmotic pressure in the colon. This suppresses the transport of water from the luminal side to the vascular side, and PHGG is thought to improve constipation.

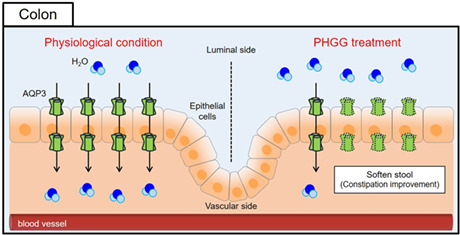

## 1 INTRODUCTION

Chronic constipation affects approximately 20% of the general population and is known to be especially prevalent in elderly individuals (De Giorgio et al., 2015; McCrea et al., 2009). As constipation is caused by a lack of water or disorders of intestinal motility, water intake and lifestyle improvements, such as eating habits and exercise, are effective ways to alleviate constipation. However, it is often difficult to relieve symptoms by improving only these lifestyle factors, and more active intervention may be needed. Various laxatives are used for the treatment of constipation, and recently functional foods have also attracted attention as a self‐medication tool. Functional foods targeting constipation include probiotics such as bifidobacteria and lactic acid bacteria and prebiotics such as dietary fiber, and these foods are currently under development and included in clinical research (Aoki et al., 2016; Liu & Zhi, 2021; Miryan et al., 2022).

Partially hydrolyzed guar gum (PHGG) is a water‐soluble dietary fiber obtained from the seed endosperms of *Cyamopsis tetragonolobus*. PHGG has various beneficial effects; it has been reported to improve hyperglycemia and hyperlipidemia (Kondo et al., 2004; Naito et al., 2016; Yasukawa et al., 2012). Moreover, there are many reports that PHGG can improve constipation (Inoue et al., 2019; Kappor et al., 2017). The improvement of constipation by PHGG has been attributed to a prebiotic effect, which is thought to be due to the alteration of the intestinal environment and the enhancement of intestinal peristalsis (Okubo et al., 1994). However, the mechanism by which PHGG improves constipation remains unclear.

In recent years, aquaporin (AQP) has been shown to be an important molecule that regulates the amount of water in the body (Verkman, 2011). AQPs are water channels that distribute throughout the human body, and 13 types, AQP0 through AQP12, have been identified. It has been found that abnormalities in AQP cause water metabolism disorder and are involved in the development of various diseases: nephrogenic diabetes insipidus (Lu, 2017; Satake et al., 2010), dry skin (Ikarashi et al., 2017, 2021), and cerebral edema (Wang et al., 2014). AQP3 is abundantly expressed in colonic mucosal epithelial cells (Silberstein et al., 1999), which regulates the water content of feces (Ikarashi et al., 2016; Laforenza, 2012). We have previously clarified that change in the AQP3 is involved in the development of constipation and diarrhea (Ikarashi et al., 2012; Ikarashi, Baba, et al., 2011; Kon et al., 2015). In this study, we investigated the role of AQP in the ameliorative effect of PHGG on constipation. Specifically, we analyzed the expression levels of various genes related to stool properties, including AQP3 in the rats fed PHGG.

## 2 MATERIALS AND METHODS

### 2.1 PHGG

PHGG was obtained by Taiyo Kagaku Co., Ltd. (Mie, Japan). The guar gum was treated with β‐endogalactomannase from an *Aspergillus niger* strain and purified to be PHGG.

### 2.2 Animals and treatment

Wistar rats (male, 8‐week‐old) were obtained from Japan SLC Co., Ltd. This animal experiment was approved by the Hoshi University (approval number; 30‐097).

Rats were allowed to freely consume a normal diet or a diet containing 5% PHGG and provided drinking water *ad libitum* for 14 days. On the 14th day, the total number of fecal pellets and fecal wet weight were determined using defecated stools from rats. The feces were dried in a freeze‐dryer for 24 hours, and the water content was calculated. In addition, the colon was removed from rats under isoflurane anesthesia.

### 2.3 Real‐time RT‐PCR

Total RNA was extracted from rat colon tissue, and cDNA was synthesized. The expression levels of each gene were detected with real‐time PCR under the following conditions: cDNA solution, SsoAdvanced SYBR green supermix (Bio‐Rad Laboratories, Hercules, CA), and mixed forward and reverse primers (Table 1). Gene expression levels were analyzed due to monitoring by the CFX Connect real‐time PCR detection system. All genes were normalized by the mRNA level of beta actin (*Actb*).

**TABLE 1:** Primer sequences

Gene	Forward (5′–3′)	Reverse (5′–3′)
*Cldn1*	ATTTCAGGTCTGGCGACATT	TAAGAGGTTGTTTTCCGGGG
*Tjp1*	GAACAGCCAACCAGTGGATA	CGGCTCAGATGACTTAGGAC
*Cdh1*	GGACCGAGAGAGTTACCCTA	CGACCTCATTCTCAAGCACT
*Aqp3*	CCCCTTGTGATGCCTCTC	CCCTAGCTGGCAGAGTTC
*Smit*	AGGAGTCCTTGGGTTGGAAC	ACTGCAACAAGGCCTCCAG
*Taut*	GTTCTGGGAGCGCAACGT	ACCGAACACCCTTCCAGATG
*Actb*	GCCACTGCCGCATCCTCTTG	CGGAACCGCTCATTGCCGAT

### 2.4 Preparation of the plasma membrane (PM)‐rich fraction

The colonic mucosa from rats was homogenized in dissecting buffer (0.3 M sucrose, 25 mM imidazole, 1 mM ethylenediaminetetraacetic acid, 8.5 μM leupeptin, and 1 μM phenylmethylsulfonyl fluoride; pH 7.2). After centrifugation (800 *g*, 4°C, 15 min), the supernatant was further centrifuged (17,000 *g*, 4°C, 30 min). The precipitates were sonicated to obtain the PM‐rich fraction (Kon et al., 2017).

### 2.5 Western blotting

After measuring the protein concentration of PM fraction, western blotting was performed. After blocking the poly vinylidene difluoride membrane, it was treated with primary antibodies, rabbit anti‐rat AQP3 antibody (Alomone Labs, Jerusalem, Israel) or mouse anti‐Na^+^/K^+^ ATPase α‐1 antibody (Merck Millipore, Darmstadt, Germany). Secondary antibodies used were sheep anti‐mouse IgG‐HRP antibody (Merck Millipore) or donkey anti‐rabbit IgG‐HRP antibody (Santa Cruz Biotechnology, Inc., Santa Cruz, CA). After the membrane was treated with detection reagents, and the bands were analyzed by luminoimage analyzer ImageQuant LAS500 (Cytiva, Tokyo, Japan).

### 2.6 Statistical analysis

The data are shown as the mean ± standard deviation (SD). Student's *t* test was performed to determine a statistically significant difference.

## 3 RESULTS

### 3.1 Effect of PHGG treatment on defecation parameters

The rats were allowed to freely consume a normal diet or a diet containing 5% PHGG for 14 days. Body weight, food intake, and defecation status were examined (Table 2).

**TABLE 2:** Body weight, food intake, and defecation parameters

	Control group	PHGG‐treated group
Body weight (g)		
Day 0	187.1 ± 8.1	185.8 ± 5.9
Day 14	254.0 ± 9.9	256.0 ± 9.4
Food intakes (g/rat/day)	17.9 ± 1.9	17.1 ± 0.5
Weight of feces (g)	0.71 ± 0.36	0.74 ± 0.29
Number of feces (counts)	4.3 ± 2.1	3.3 ± 1.7
Fecal water contents (%)	100.0 ± 4.7	120.0 ± 9.9*

*Note*: Normal rats were allowed to freely consume a normal diet or a diet containing 5% PHGG for 14 days. The body weight, food intake, weight of feces, numbers of feces, and fecal water contents were examined (mean ± SD, *n* = 7, *p < .05).

The body weight and food intake of rats treated with PHGG were almost the same as those of rats in the control group. The total weight of fecal pellets and the total number of fecal pellets were not changed by the administration of PHGG. However, the water content in feces of the PHGG‐treated group was significantly increased by approximately 1.2 times.

Based on these results, PHGG increased fecal water content without affecting the total weight and number of fecal pellets.

### 3.2 Effect of PHGG on the expression of genes involved in tight junctions and adherence junctions in the colon

In the colon, water is transported from the luminal side to the vascular side by the paracellular and transcellular pathways (Fischbarg, 2010; Hill & Shachar‐Hill, 2006). In the paracellular pathway, water is transported via tight junctions and adherence junctions (Suzuki, 2013). Therefore, we analyzed the mechanism by which PHGG increases fecal water content, focusing on claudin‐1 (CLDN1) and tight junction protein‐1 (TJP1), which are components of tight junctions, and cadherin‐1 (CDH1), which is a component of adherence junctions.

**FIGURE 1:**
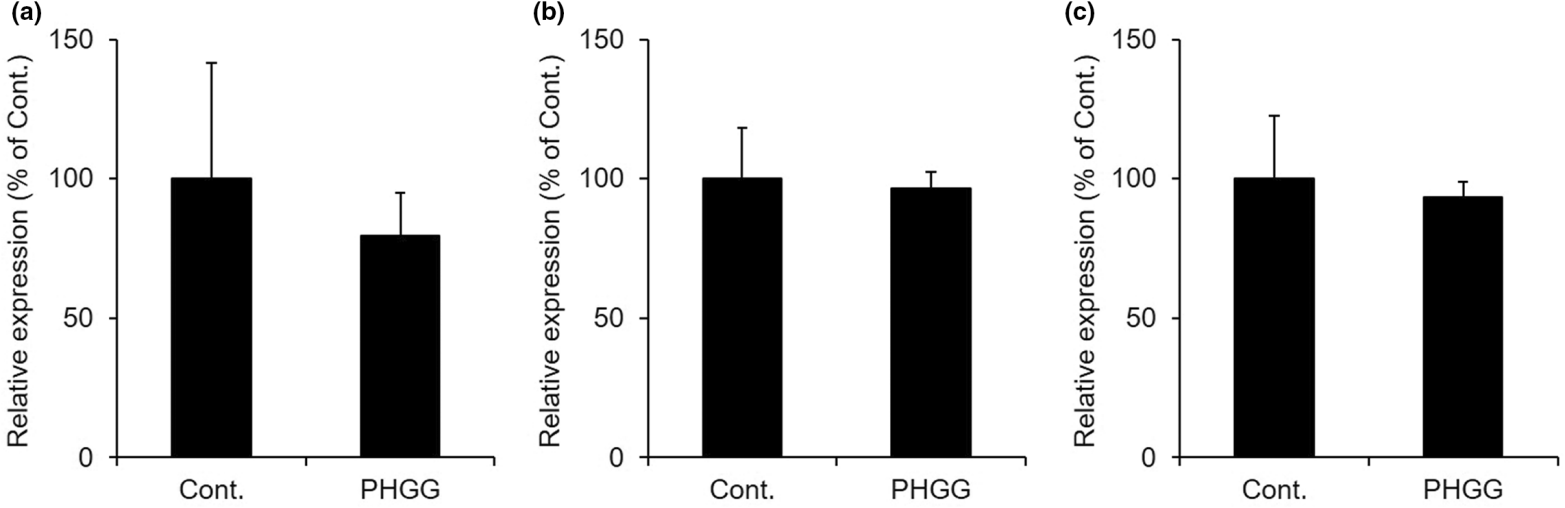
The *Cldn1*, *Tjp1*, and *Cdh1* mRNA expression levels in the colon. Normal rats were allowed to freely consume a normal diet or diet containing 5% PHGG for 14 days. The *Cldn1* (a), *Tjp1* (b), and *Cdh1* (c) mRNA expression levels in the colon were measured by real‐time RT‐PCR (mean ± SD, *n* = 7).

The *Cldn1* mRNA expression level in the colon of the PHGG‐treated group was almost the same as that of the control group (Figure 1a). Similarly, there were no differences in the expression levels of *Tjp1* and *Cdh1* between the PHGG‐treated group and the control group (Figure 1b, c).

Based on these findings, tight junctions or adherence junctions in the colon do not seem to be related to the increase in fecal water content during the PHGG treatment.

### 3.3 Effect of PHGG on AQP3 expression in the colon

AQP3 is predominantly expressed in the colon and is involved in water transport through the transcellular pathway. As the osmotic pressure on the luminal side is lower than that on the vascular side in the colon under physiological conditions, water is transported from the luminal side to the vascular side through AQP3, and stool is concentrated (Ikarashi et al., 2016). Therefore, we investigated the possibility that the PHGG‐induced increase in fecal water content was caused by changes in AQP3 expression.

**FIGURE 2:**
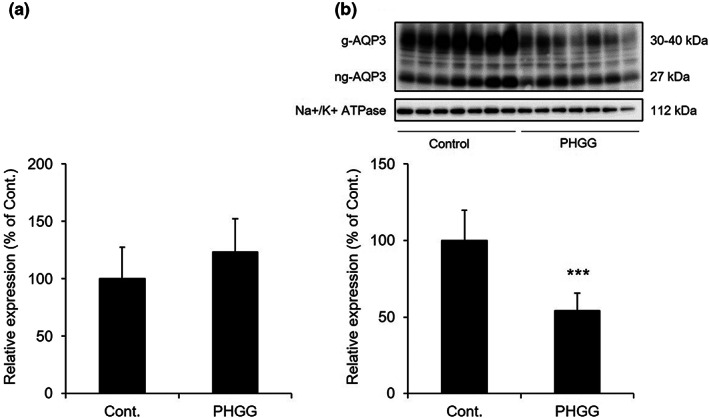
The mRNA and protein expression levels of AQP3 in the colon. Normal rats were allowed to freely consume a normal diet or diet containing 5% PHGG for 14 days. (a) The *Aqp3* mRNA expression in the colon was measured by real‐time RT‐PCR. (b) The AQP3 protein expression level in the colon was measured by western blotting (mean ± SD, *n* = 7, ***p < .001).

**FIGURE 3:**
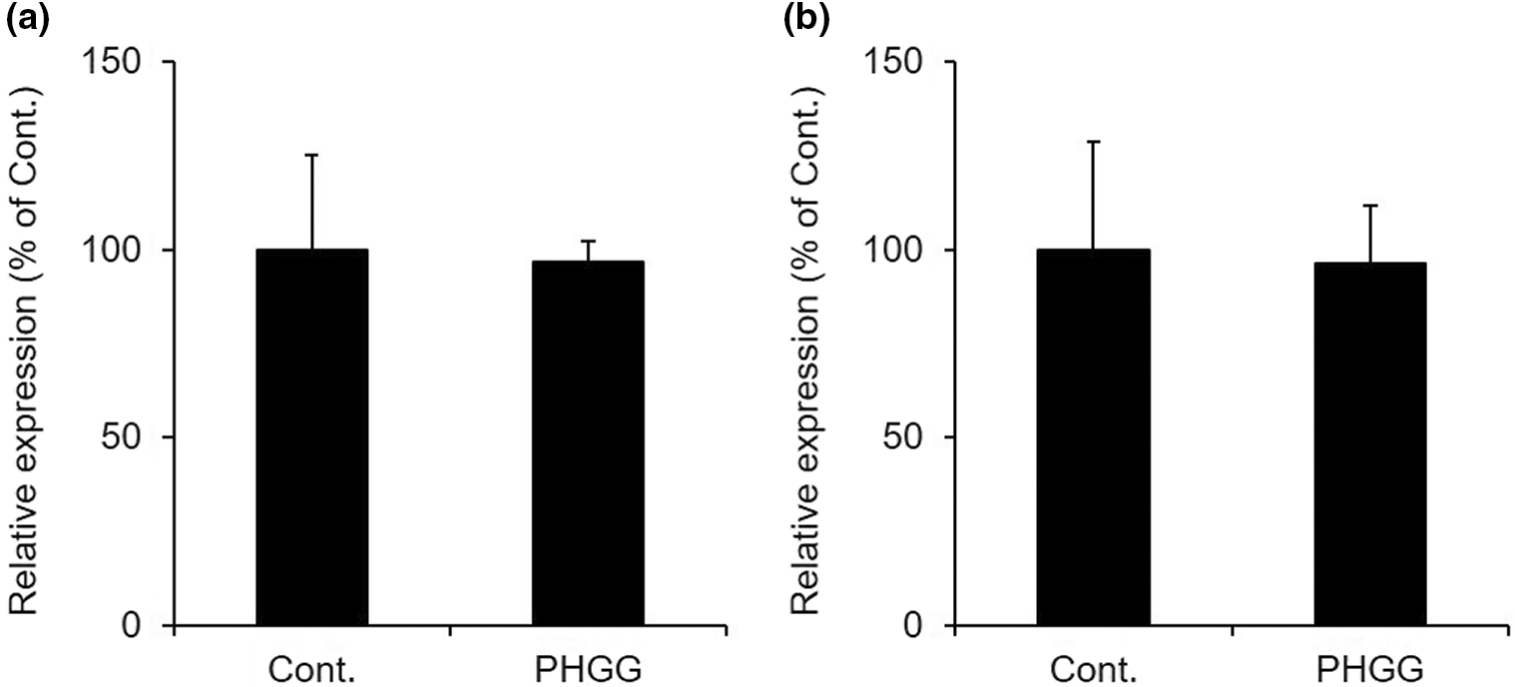
The mRNA expression levels of *Smit* and *Taut* in the colon. Normal rats were allowed to freely consume a normal diet or a diet containing 5% PHGG for 14 days. The *Smit* (a) and *Taut* (b) mRNA expression levels in the colon were measured by real‐time RT‐PCR (mean ± SD, *n* = 7).

There was no difference in the mRNA expression level of *Aqp3* between the PHGG‐treated group and the control group (Figure 2a). AQP3 protein expression was detected as deglycosylated AQP3 (27 kDa) and glycosylated AQP3 (30–40 kDa) by western blotting (Silberstein et al., 1999; Spector et al., 2002). The AQP3 protein expression level was significantly decreased in the PHGG‐treated group (Figure 2b). For the purpose of investigating the intraluminal osmotic pressure that regulates the water transport direction by AQP3, we examined the mRNA expression levels of sodium *myo*‐inositol cotransporter (*Smit*) and taurine transporter (*Taut*) as genes that reflect change in osmotic pressure in colonic lumen (Ikarashi, Ushiki, et al., 2011; Minami et al., 1996), were measured. No difference was observed between the PHGG‐treated group and the control group (Figure 3).

These data suggest that the increase in fecal water content during PHGG treatment may be related to the decrease in the AQP3 expression in the colon.

## 4 DISCUSSION

Constipation, a defecation disorder, is not directly life threatening, therefore its treatment is often neglected. However, constipation interferes with daily life and mental health (Belsey et al., 2010; Sun et al., 2011). In this study, to elucidate the mechanism by which the water‐soluble dietary fiber PHGG improves constipation, we investigated the action of PHGG on defecation status, focusing on the colonic water channel AQP3.

In this study, rats were fed a normal diet containing 5% PHGG for 14 days (Okamura et al., 2022; Takagi et al., 2016). The defecation frequency and volume of the rats in the PHGG treatment group were not different from those of rats in the control group. In contrast, the fecal water content in the PHGG‐treated group was significantly increased (Table 2). There was no change in food intake between the PHGG‐treated group and the control group throughout the administration period. In addition, there was no difference in body weight between the two groups at the end of the treatment (Table 2). These findings suggest that the increase in fecal water content by PHGG treatment was not caused by toxicity, and there is no problem with the dose. Furthermore, the above findings suggested that PHGG may have increased the fecal water content and softened the feces without affecting peristaltic movement.

Orally ingested water and digestive fluids secreted from the small intestine are absorbed while passing through the gastrointestinal tract, eventually concentrating stool in the colon. We analyzed the mechanism underlying the PHGG‐induced increase in fecal water content, focusing on genes important for water transport in the colon. Colonic epithelial cells have strong tight junctions and adherence junctions, and the disruption of these junctions causes diarrhea (Schneider et al., 2010; Van Sebille et al., 2018; Yu et al., 2017). Therefore, we investigated the effects of PHGG on the expression levels of CLDN1 and TJP1, constituent proteins of tight junctions, and the expression level of CDH1, a constituent protein of adhesion junctions. The expression levels of *Cldn1*, *Tjp1*, and *Cdh1* in the colon of the PHGG‐treated group were almost the same as those of the control group (Figure 1). Therefore, it is unlikely that PHGG acted on tight junctions or adhesion junctions in the colon.

As a route for the water transport through the cell in the colon, the expression level of AQP3 is an important. The AQP3 protein expression was significantly decreased in the PHGG‐treated group (Figure 2). Additionally, there were no differences in the levels of *Smit* and *Taut* (Ikarashi, Ushiki, et al., 2011; Minami et al., 1996) between both groups (Figure 3). These findings suggested that PHGG did not change the osmotic pressure in the colonic lumen, and that water was transported from the luminal side to the vascular side in the normal physiological absorption direction. The above results indicated that the increase in fecal water content by PHGG may have been caused by the decrease in the expression of colon AQP3.

Why did PHGG treatment decrease the AQP3 expression in the colon? In previous reports, AQP3 expression decreases by inflammation (Horie et al., 2009; Ikarashi, Baba, et al., 2011). However, the results showed no change in the expression levels of inflammatory cytokines (tumor necrosis factor‐α, interleukin‐1β, and interleukin‐6) in the colon after treatment with PHGG (data not shown). Therefore, inflammation does not seem to be related to decrease in colonic AQP3 caused by PHGG treatment.

Diarrhea is caused by impaired absorption or altered the synthesis of bile acids, which is called bile acid diarrhea. Recently, it has been reported that when bile acid diarrhea develops, *Aqp3* mRNA is not affected, but AQP3 protein is decreased (Yde et al., 2016). On the other hand, PHGG is known to change the amount of gut microbiota and its metabolites in the colon, such as bile acids and short‐chain fatty acids (Kovacs et al., 2002; Levrat‐Verny et al., 2000; Takayama et al., 2021). Although the mechanism by which PHGG decreases the expression of AQP3 is speculative, since the low gastrointestinal absorption of PHGG, AQP3 was considered to be decreased by the following mechanism. Oral administration of PHGG changes the gut microbiota and thereby the amount of gut microbiota metabolites changes, decreasing AQP3. The details of this mechanism will be clarified in the future.

**FIGURE 4:**
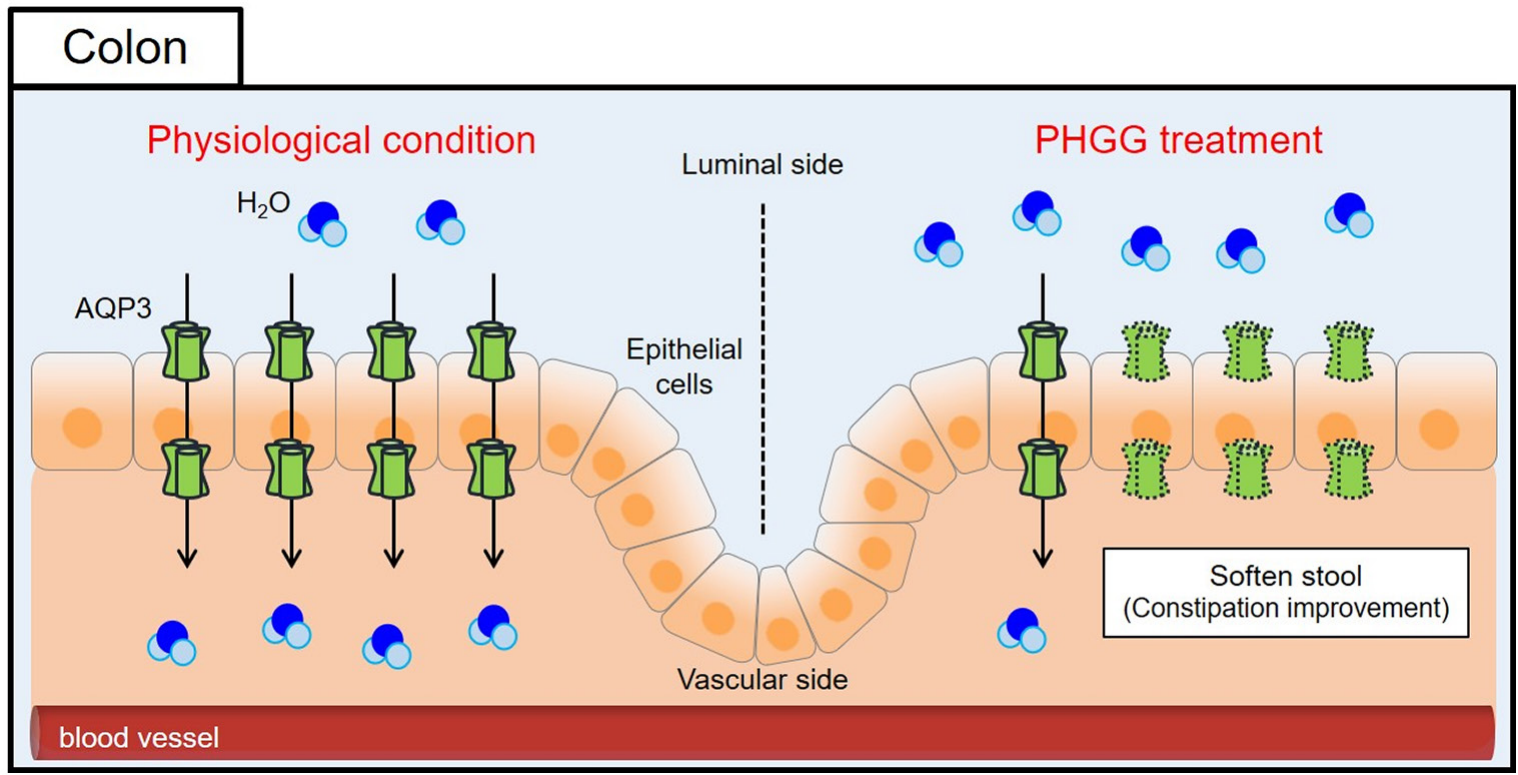
Effect of PHGG on the colonic AQP3

In summary, we found that PHGG decreases the AQP3 expression level without changing the osmotic pressure in the colon. This suppresses water movement from the luminal side to the vascular side, thus softening the stool and PHGG improves constipation (Figure 4). In the future, scientific evidence supporting the use of prebiotics can be clarified by elucidating the mechanism by which AQP3 expression is decreased by PHGG.

## FUNDING INFORMATION

This study was funded by the Lotte Shigemitsu Prize and Taiyo Kagaku Co., Ltd.

## CONFLICT OF INTEREST

M.O. and Z.Y. are employees of Taiyo Kagaku Co., Ltd. Neither the external organization nor the funding agency participated in research design or no competing interests. The other authors (R.K., N.I., K.O., H.S., and J.K.) declared that the study was conducted in the absence of financial relationships that could be constructed as potential conflicts of interest.

## Data Availability

Data will be made available on request.
